# Case report: Transcatheter arterial embolization in a newborn with cervical rapidly involuting congenital hemangioma and Kasabach–Merritt phenomenon

**DOI:** 10.3389/fped.2023.1073090

**Published:** 2023-02-24

**Authors:** Ying-Hsuan Peng, Ming-Chih Lin, Wei-Li Liu, Sheng-Ling Jan

**Affiliations:** ^1^ Department of Pediatrics, Chung Shan Medical University Hospital, Taichung, Taiwan; ^2^ Department of Pediatrics, School of Medicine, Chung Shan Medical University, Taichung, Taiwan; ^3^ Department of Pediatrics, Children's Medical Center, Taichung Veterans General Hospital, Taichung, Taiwan; ^4^ Department of Pediatrics, Dalin Tzu Chi Hospital, Chiayi, Taiwan; ^5^ Department of Pediatrics, School of Medicine, National Yang-Ming University and Kaohsiung Medical University, Hsinchu and Kaohsiung, Taiwan

**Keywords:** congenital hemangioma, Kasabach–Merritt phenomenon, coagulopathy, transarterial embolization, infants

## Abstract

Congenital hemangiomas (CHs) are rare vascular tumors and do not exhibit progressive postnatal growth. The incidence is less than 3% of all hemangiomas. Most CHs have a favorable prognosis; however, the Kasabach–Merritt phenomenon (KMP) is a rare but life-threatening complication in CHs that requires aggressive treatment. Medical treatments with corticosteroids and interferon have been suggested. Surgical resection can be considered for the treatment of complicated CHs in medically resistant lesions. Vascular embolization could be an alternative method if surgery is not considered feasible. Herein, we report a case of a 9-day-old newborn who underwent arterial embolization for a CH with KMP, combined with sirolimus treatment, and the outcome was favorable. The hemangioma completely regressed by 3 months and rapidly involuting congenital hemangioma (RICH) was diagnosed. Our successful experience with treating RICH associated with KMP revealed that RICH can have potentially serious complications although they usually resolve rapidly after birth without treatment. Surgical resection is considered to be the standard method for the treatment of medically resistant vascular tumors, but it is difficult to perform during the active phase of KMP due to acute bleeding and severe coagulopathy. Arterial embolization is feasible and can be used as an alternative to surgical resection, even in small babies.

## Case report

**Figure 1:**
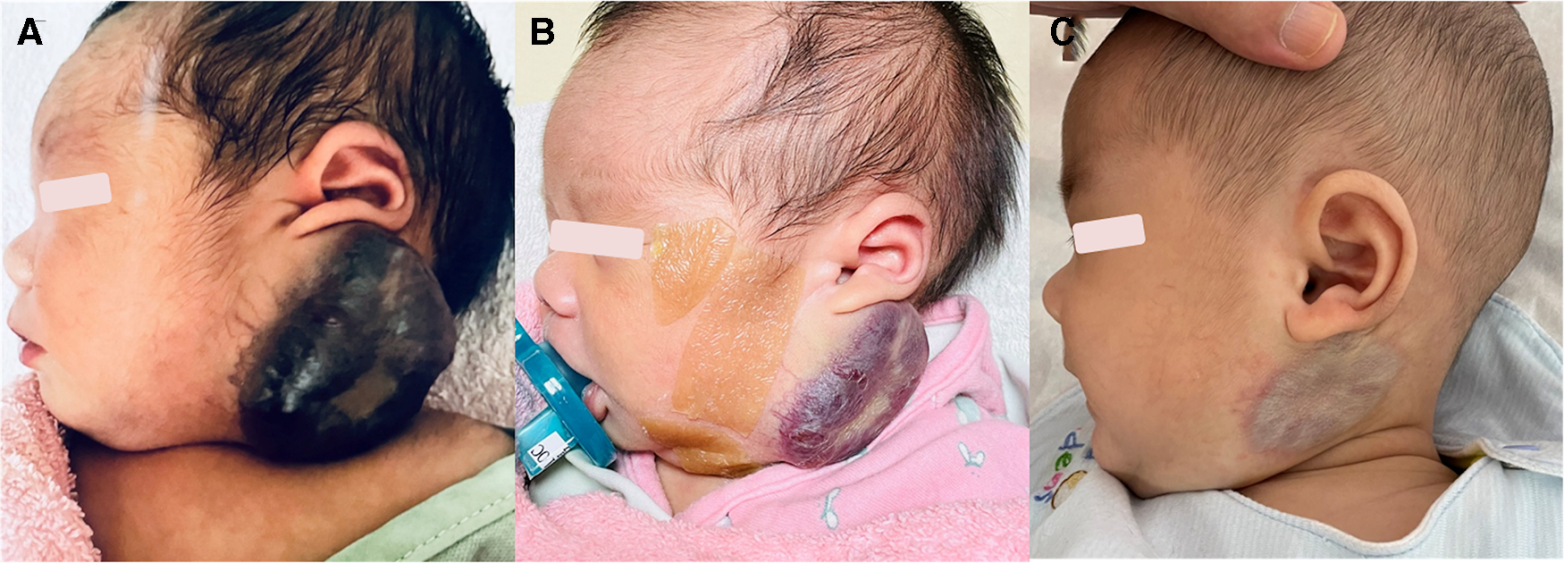
(**A**) A purple, telangiectatic, centrally depressed mass with pale margins was found behind the left ear at birth. (**B**) The mass shrank 2 weeks after arterial embolization. (**C**) The mass had almost completely resolved by 3 months of age, leaving a telangiectatic scar with halo margins.

**Figure 2:**
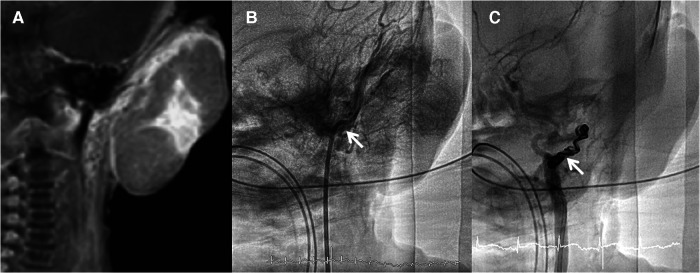
(**A**) Magnetic resonance imaging showed a well-defined margin, vascular-rich lesion with a central scar. (**B**) Selected left common arteriography demonstrated a clear boundary, uniform staining mass with a feeder artery from the internal carotid artery to the mass without collateral artery to adjacent organs (arrow). (**C**) Repeat arteriography after arterial embolization using multiple coils shows no residual shunt (arrow).

The patient was a male newborn from the first pregnancy of a clinically healthy woman. He was born at 38 + 6 weeks of gestational age by normal spontaneous delivery, weighing 2,890 g. His mother received regular prenatal examinations revealing no specific abnormality. There was a large, firm, immovable oval mass approximately 6 cm × 5 cm × 2 cm in diameter behind his left ear, with a vibrating surface on palpation, suggesting a possible fast-flowing vascular tumor. The lesion was purple in color and had a telangiectatic appearance with a central depression and pale margins (Figure 1A). He was initially treated with propranolol combined with simple occlusive dressings containing epinephrine; however, the hemangioma became engorged with massive bleeding from the sixth day of life. Laboratory data showed Kasabach–Merritt phenomenon (KMP) with thrombocytopenia (platelet of 17,000/μL), coagulopathy (international normalized ratio of prothrombin time was 2.04 and activated partial thromboplastin time was greater than 150 s), and hypofibrinogenemia (fibrinogen of 152.1 mg/dL). Magnetic resonance imaging (MRI) on the seventh day of life revealed a well-defined margin, T2 high-intensity signals, flow voids, a vascular-rich lesion with a central scar, no intracranial extension or involvement of adjacent tissues, and confirmed the presence of a hemangioma (Figure 2A). Biopsy was not performed due to the risk of bleeding from an associated consumptive coagulopathy. After the diagnosis of Kasabach–Merritt phenomenon was made, propranolol was shifted to sirolimus 0.8 mg/m^2^ twice daily. Platelet was transfused for thrombocytopenia. Fresh frozen plasma and cryoprecipitate were given due to coagulopathy. However, the active bleeding persisted. Thrombocytopenia and coagulopathy cannot be corrected under medical treatment. Platelet decreased to 14,000/μL. Hemoglobin dropped from 14 to 7.4 mg/dL. Pediatric surgeon was consulted for the possibility of surgery, but he hesitated because of the higher risk during the acute phase of KMP. Therefore, transcatheter arterial embolization was performed at 9 days of age and 3.05 kg weight. Selective left common carotid arteriography demonstrated a clear boundary, uniform parenchymal staining mass with a single large feeder artery from the internal carotid artery (Figure 2B). A 4F delivery catheter (Amplatzer Judkins Right catheter, Abbott Medical, MN, United States) was advanced over a 0.018″ Terumo guide wire into the feeding artery of the mass lesion. A total of three Cook Embolization Coils, two 3 mm × 4 cm, one 3 mm × 5 cm, and one 3/2 mm × 2 cm Cook Tornado Embolization Microcoil (Cook Medical, Bloomington, IN, United States), were selected for embolization of the feeding artery. Repeat arteriography showed no obvious residual shunt after embolization (Figure 2C). After transcatheter embolization, the bleeding stopped. Moreover, there was no transcatheter complications noted including renal impairment or femoral vascular damage. Laboratory data revealed neither thrombocytopenia nor coagulopathy 1 week later. No more blood transfusion was needed. Due to stable condition, he was discharged from the hospital on day 18 (Figure 1B). At 3 months, the hemangioma had almost completely regressed, leaving a halo-shaped telangiectatic scar (Figure 1C), and then sirolimus was shifted to propranolol. He received regular follow-up at outpatient department. The scar became lighter and lighter gradually without hemangioma recurred. Propranolol was tapered and discontinued 6 months later. Eventually, rapidly involuting congenital hemangioma (RICH) was diagnosed and cured successfully by transcatheter arterial embolization. Medical treatment do not require in a long time.

## Discussion

Infantile hemangiomas (IHs) with strawberry-like bright red and ridged appearance are common, occurring in 1%–2% of newborns (1). Typical IHs rapidly grow 1 year after birth, followed by slow complete involution in subsequent years. Congenital hemangiomas (CHs) are often clinically and histopathologically distinguishable from IHs. CHs are rare, accounting for less than 3% of all hemangiomas (2). CHs are fully developed at birth and do not proliferate further (3). The diagnosis of CHs is based on the combination of clinical, histological, and imaging features and is confirmed by subsequent changes of the tumor. CHs are usually diagnosed as high-flow parenchymal, violaceous, and telangiectatic masses. CHs are mainly classified into two types: RICH and non-involuting congenital hemangioma (NICH). RICH and NICH share many similarities in appearance, location, size, sex distribution, and imaging studies, but both have different regressive changes and overlapping histological appearances. RICH, the most common type of CH, tends to have accelerated regression after birth, so lesions usually resolve completely by 6–14 months, leaving atrophic skin. NICH does not resolve, defined as no change in size or color by 6 months, usually requiring surgical excision (4).

CHs, especially RICHs, can look very much like Kaposiform hemangioendothelioma (KHE), particularly in the neonatal period and should be carefully differentiated (5). KHE is a rare vascular tumor of infancy that presents as a locally infiltrating subcutaneous violaceous mass. Spontaneous involution is very rare in KHE and usually requires multimodal treatment. KMP, a severe, life-threatening consumptive coagulopathy with profound thrombocytopenia and hypofibrinogenemia with elevated coagulation activation markers, is a common complication of KHE and occasionally occurs in CHs. KMP is currently thought to occur in approximately 42%–71% of KHE and less than 10% of CHs (3, 6, 7). A comparison of the two common infantile vascular tumors associated with KMP is shown in Table 1 (3–6, 8).

**Table 1:** Comparison of two common infantile vascular tumors associated with KMP (3–6, 8).

	RICH	KHE
Incidence	<3–6/10,000 (4)	<1/10,000 (6)
Sex distribution	Female = male (4)	Slight male predominance (6)
**Clinical presentation**		
Location	Head/neck = extremities	Extremities > trunk or head/neck, 12% extracutaneous
Size	1–15 cm	0.9–7 cm
Appearance	Raised, round or ovoid, violaceous tumor, covered with telangiectases, surrounding pale rim, central ulcer/scar	Slightly raised, erythematous papule, plaque, or nodule to an indurated, purple, and firm tumor
Complications	Occasional; ulceration with bleeding, compression of vital structures, congestive heart failure	Common; local invasive features, compression of vital structures, lymphedema
KMP	Occasional, less than 10% (3)	Frequent, about 42%–71% (6)
**Diagnostic modalities**		
Ultrasonography	Echogenic, well-defined vascular parenchymal mass with fast-flow, occasional venous lakes/ectasia	Heterogeneous echogenicity, ill-defined vascular mass with infiltrative pattern, rare venous lakes/ectasia
MRI	Well-defined margins, T2 hyperintense masses with high-flow vessels, flow voids, and without adjacent destruction	Ill-defined margins, multiplanar, diffuse enhancement, T1 isointensity relative to adjacent muscle, T2 hyperintensity, occasional flow voids
Angiography	Clear boundary, uniform staining, distributed on one side of the normal artery, usually 1–4 feeder arteries, and the diameter is proportional to the tumor size	Obscure boundary, uneven staining, surrounding the normal artery, fine and multifeeder arteries, and the diameter is not proportional to the tumor size
Biopsy	Circumscribed small lobules of capillaries lined by endothelial cells and surrounded by fibrous tissue, absent staining for the lymphatic markers and glucose transporter-1 (GLUT-1)	Coalescing nodules of spindled endothelial cells, slit-like or crescentic vessels containing hemosiderin, positive staining for the lymphatic markers, negative for glucose transporter-1 (GLUT-1)
**Treatment and prognosis**		
Treatment	Most (about 90%) do not require therapy and propranolol is ineffective. corticosteroids, interferon-α, surgical resection	Propranolol, corticosteroids, vincristine, sirolimus, interferon-α, surgical resection. Needs combination therapy if with KMP
Embolization	Feasible technique to embolize for larger and fewer feeding vessels	Challenging technique to embolize for fine and multifeeding vessels
Prognosis	Most fully regress by 12 months, mortality is rare (6)	Rare spontaneous involution, most progressive enlargement, 30% mortality (6)

KHE, kaposiform hemangioendothelioma; KMP, Kasabach–Merritt phenomenon; MRI, magnetic resonance imaging; RICH, rapidly involuting congenital hemangioma.

Since most RICHs usually resolve rapidly after birth, most affected cases do not require treatment and only need to be kept in observation. However, RICH can have potentially serious complications, such as ulceration with bleeding, vital organ compression, KMP, and congestive heart failure. Treatment of complicated RICH includes assessment of hemodynamic status, wound care, pharmacologic therapy, and surgery or embolization. Compared to IHs, propranolol treatment is now known to be ineffective for CHs (9). Corticosteroid and interferon alfa-2a are used for some dangerous lesions (3). Surgical resection is indicated for persistent ulceration and bleeding, sepsis, hemodynamic instability, or high-output cardiac failure in medically resistant cases. RICH complicated by KMP can be mild and transient, but there have also been reports of RICH complicated by severe and life-threatening KMP, including our case (3). As the mortality rate of KMP is as high as 8%–24% (10), accurate diagnosis of KMP and prompt treatment are very important. Due to the rarity of cases of RICHs complicated with KMP (11), there are currently no standard treatment guidelines for RICH complicated with KMP. Treatment is based primarily on expert opinion as well as treatment experience with a small number of KMP case reports and case series (12, 13). Supportive treatment should be provided first to prevent the patient's condition from getting worse, followed by curative therapy for the underlying vascular tumor. Fresh frozen plasma or cryoprecipitate is used to correct coagulopathy. Platelets are reserved for active bleeding during or before surgery because it will exacerbate mass bleeding and the trapping of platelets in the tumor (6, 13). Surgical resection is considered to be the standard method for the treatment of vascular tumors, but it is difficult to perform during the active phase of KMP due to acute bleeding and severe coagulopathy. Instead, technical difficulty is not the main limitation of transcatheter arterial embolization nowadays. It is very straightforward for experienced pediatric interventional cardiologists and radiologists. Advantages of arterial embolization include lower risk of anesthesia, smaller wound, and shorter recovery time. We should still pay attention to avoid transcatheter complications such as contrast allergy, renal impairment, thrombus formation, or vascular injury during the procedure.

In our case, embolization was initially performed because surgery was considered too risky. On the other hand, pharmacologic therapy takes too long to correct coagulopathy with active bleeding. Therefore, transcatheter arterial embolization may be a feasible therapy for the treatment of RICH complicated by KMP, because RICH is characterized by larger and fewer feeder vessels. In contrast, in neonates and young infants with KHE, access to very small and numerous feeder arteries remains technically more difficult (6, 8). In this case, a 9-day-old neonate with RICH underwent arterial embolization in combination with sirolimus for a rare but life-threatening complication of KMP with good outcomes. Our successful experience with treating RICH associated with KMP revealed that RICH can have potentially serious complications although they usually resolve rapidly after birth without treatment. Surgical resection is considered to be the standard method for the treatment of medically resistant vascular tumors, but it is difficult to perform during the active phase of KMP due to acute bleeding and severe coagulopathy. Arterial embolization is feasible and can be used as an alternative to surgical resection, even in small babies.

## Data Availability

The original contributions presented in the study are included in the article/Supplementary material, further inquiries can be directed to the corresponding author.
